# Complete Genome Sequence of Salmonella enterica Serovar Typhi Strain ISP2825

**DOI:** 10.1128/MRA.00804-21

**Published:** 2021-10-14

**Authors:** Gi Young Lee, Jeongmin Song

**Affiliations:** a Department of Microbiology and Immunology, Cornell University, Ithaca, New York, USA; University of Maryland School of Medicine

## Abstract

Salmonella enterica serovar Typhi ISP2825, isolated in 1983 from a Chilean patient, is one of the major *S.* Typhi strains used for research, along with strains Ty2, CT18, and H58. The complete genome sequence of ISP2825, consisting of a 4,774,014-bp circular chromosome, will help us understand typhoid pathogenesis and evolution.

## ANNOUNCEMENT

Salmonella enterica serovar Typhi is the causative agent of the life-threatening systemic disease typhoid fever, which is a major cause of infection-mediated morbidity and mortality in countries of endemicity. Humans are the only known host of *S.* Typhi. Despite its narrow host specificity, *S.* Typhi has remained a highly successful pathogen since its emergence (1). Typhoid vaccines are available, but their efficacies are imperfect (2). Increasing antibiotic resistance of *S.* Typhi has been reported. Recently, multidrug-resistant (MDR) and extensively drug-resistant (XDR) strains have become the dominant *S.* Typhi variants (3).

**FIG 1:**
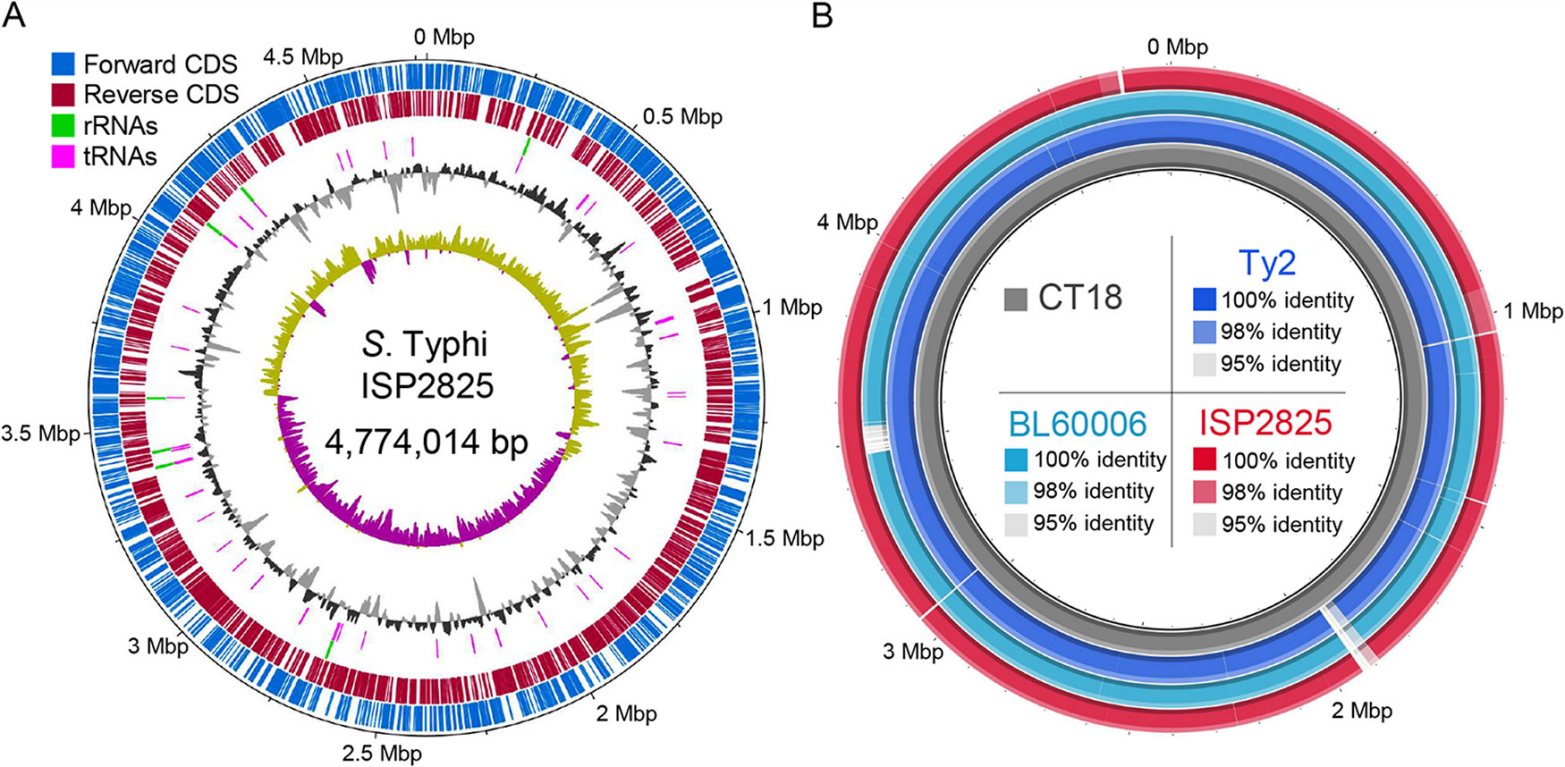
Circular maps summarizing features of *S.* Typhi ISP2825 (A) and comparative analysis of ISP2825, Ty2, BL60006, and CT18 (B). The ISP2825, Ty2, and BL60006 genomes were compared to the genome of CT18. Figure 1A and B were generated using DNAPlotter in Artemis (https://github.com/sanger-pathogens/Artemis) and BRIG v0.95 (https://sourceforge.net/projects/brig), respectively. CDS, the coding region of genes; BL60006, an XDR *S.* Typhi strain isolated from a Pakistani patient belonging to the H58 clade.

There are four major *S.* Typhi strains extensively used for research that have helped advance our understanding of typhoid pathogenesis and evolution: Ty2 (4), CT18 (5), H58 (6), and ISP2825 (7–24). Complete genome sequences of these strains are available, except for ISP2825. Here, we report the complete genome sequence of *S.* Typhi ISP2825. In brief, *S.* Typhi ISP2825 was cultured in 2 ml Luria-Bertani broth (LB) overnight at 37°C, and its genomic DNA was extracted using the DNeasy blood and tissue kit (Qiagen, Germany), without processing additional fragmentation and size selection. The genomic DNA quality and quantity were monitored using a NanoDrop 2000 spectrophotometer (Thermo Fisher Scientific, USA) and a Qubit 4 fluorometer (Thermo Fisher Scientific). A genomic DNA library was prepared using a SQK-LSK110 ligation sequencing kit (Oxford Nanopore Technologies [ONT], UK), followed by sequencing with two MinION Flongle flow cells (R9.4.1) using MinKNOW v21.06.0 (ONT). The combined raw reads from two Flongle flow cells were used for base calling using Guppy v5.0.11 (ONT). Fastq files having Q scores of ≥8 were collected, filtered using NanoLyse v1.2.0 (25) to remove internal positive-control (DNA CS) sequences, and assessed for data quality using NanoPlot v1.38.1 (25) (Table 1). Next, the filtered reads were assembled using Flye v2.8.3 (26), with five iterations of the polishing step (–iterations 5), generating a single circular draft assembly. The resulting assembly was aligned using bwa v0.7.17 (https://github.com/lh3/bwa) and polished sequentially using Racon v1.4.22 (-m 8 -x -6 -g -8 -w 500) (27), Medaka v1.4.3 (https://github.com/nanoporetech/medaka) (-m r941_min_hac_g507), and Homopolish v0.2.3 (-s bacteria.msh -m R9.4.pkl) (28). The quality of the genome assembly was evaluated using QUAST v5.1.0 (29). Fasta_shift (https://github.com/b-brankovics/fasta_tools) was used to set the start position of the polished assembly according to that of *S.* Typhi CT18 (GenBank accession number GCF_000195995.1) and Ty2 (GCF_000007545.1). The complete genome sequence was annotated using the NCBI Prokaryotic Genome Annotation Pipeline (PGAP) (30), and its predicted serotype and antimicrobial resistance profiles were computed using SeqSero2 v1.2.1 (31) and AMRFinderPlus v3.10.5 (32), respectively (Table 1). Default options were used unless otherwise indicated. The complete genome sequence of ISP2825 consists of a 4,774,014-bp circular chromosome containing 4,688 genes, 4,310 protein coding genes, 267 anticipated pseudogenes, 22 rRNAs, and 79 tRNAs (Table 1). Overall, ISP2825 is similar to the other *S.* Typhi strains sequenced; notable differences are indicated in Fig. 1.

**TABLE 1:** Features of the complete genome sequence of *S.* Typhi ISP2825

Feature(s)	Value(s)
Filtered raw reads (Q score, ≥8)	
Total no. of reads	422,174
Total length (bp)	2,431,036,002
*N*_50_ (bp)	11,788
Complete genome	
Structure	Circular
Total length (bp)	4,774,014
G+C content (%)	52
Avg coverage (×)	511
Total no. of genes	4,688
No. of protein coding genes	4,310
No. of anticipated pseudogenes	267
No. of CRISPR arrays	1
RNA genes	
No. of rRNAs (5S, 16S, 23S)	8, 7, 7
No. of tRNAs	79
No. of noncoding RNAs	10
Computed antimicrobial resistance profile	None
Computed serotype	9:d:- (Typhi)

### Data availability.

The complete genome sequence of *S.* Typhi ISP2825 has been deposited at GenBank under accession number GCF_019645915.1 or CP080960.1, BioProject accession number PRJNA753482, BioSample accession number SAMN20695325, and Sequence Read Archive (SRA) accession number SRR15411315.
